# Female genital schistosomiasis as an evidence of a neglected cause for reproductive ill-health: a retrospective histopathological study from Tanzania

**DOI:** 10.1186/1471-2334-6-134

**Published:** 2006-08-23

**Authors:** Britta Swai, Gabriele Poggensee, Sabina Mtweve, Ingela Krantz

**Affiliations:** 1 Department of Pathology, Kilimanjaro Christian Medical Centre, Moshi, Tanzania; 2 Department of Infectious Disease Epidemiology, Robert Koch Institute, Seestraße 10, 13353 Berlin, Germany; 3 Community Health Department, Kilimanjaro Christian Medical Centre, Moshi, Tanzania; 4 Skaraborg Institute for Research and Development, Skövde, Sweden; 5 Department of Public Health and Clinical Medicine, Epidemiology, Umeå University, Sweden

## Abstract

**Background:**

Schistosomiasis affects the reproductive health of women. Described sequelae are ectopic pregnancy, infertility, abortion, and cervical lesions and symptoms mimicking cervical cancer and STIs. There are indications that cervical schistosomiasis lesions could become co-factors for viral infection such as HIV and HPV.

**Methods:**

In a retrospective descriptive histopathological study clinical specimens sent between 1999 and 2005 to the pathology department of a consultant hospital in Tanzania were reviewed to analyse the occurrence and features of schistosomiasis in female genital organs.

**Results:**

During the study period, schistosomiasis was histopathologically diagnosed in 423 specimens from different organs (0.7% of all specimens examined in the study period), out of those 40% were specimens from female and male organs. The specimens were sent from 24 hospitals in 13 regions of mainland Tanzania. Female genital schistosomiasis was diagnosed in 125 specimens from 111 patients. The main symptoms reported were bleeding disorders (48%), ulcer (17%), tumor (20%), lower abdominal pain (11%) and infertility (7%). The majority of cases with genital schistosomiasis were diagnosed in cervical tissue (71 cases). The confirmation of cervical cancer was specifically requested for 53 women, but the diagnosis could only be verified for 13 patients (25%), in 40 cases only severe cervical schistosomiasis was diagnosed. Vulval/labial schistosomiasis was seen in specimens from young women. Infertility was reported in four patients with schistosomiasis of the Fallopian tubes.

**Conclusion:**

Genital schistosomiasis adds to the disease burden of women in all age groups. Pathological consequences due to the involvement of different genital organs can be damaging for the affected women. Clinical unawareness of genital schistosomiasis can lead to misdiagnosis and therefore false and ineffective therapy. In endemic areas cervical schistosomiasis should be considered as differential diagnosis of cancer.

## Background

Ill-health due to reproductive health problems and sexually transmitted infections (STIs) is mostly both preventable and remediable to its nature. These illnesses, notwithstanding, account for almost one fifth of the health burden in women [1]. Many go unrecognised, since, as could be expected, the heaviest toll befalls women in the poorest countries where services are limited. Sexual and reproductive health are affected by socio-cultural factors; both at individual and societal level stigmatization and shame are deeply engrained factors influencing whatever actions are taken against these diseases and their symptoms.

Although both health professionals and the public are aware of the dismal statistics for STIs, few seem to realize that there are other infectious agents than bacteria and virus that can havoc and complicate reproductive health. Symptoms and signs associated with a STI are not uncommon in schistosomiasis, a parasitic helminth disease afflicting around 250 million people, again in the poorest countries where services are limited [2]. More than is usually acknowledged schistosomiasis affects the reproductive organs of women. Female genital schistosomiasis was described for the first time in a young Egyptian woman more than 100 years ago [3], but still awaits its proper place among the public health explanatory and target factors for sexual and reproductive ill-health. Schistosomiasis and its association with ectopic pregnancy, infertility, abortion, and cervical lesions similar to STI or cervical cancer have been described in many case reports [4]. Cervical schistosomiasis causes damages of the epithelium and these lesions, if manifest before sexual debut, could become considerable co-factors for the transmission of viral infections such as HIV and HPV infections in early ages [5,6].

Systematic histopathology studies made by pathologists and gynecologists in schistosomiasis endemic areas in the 1970s and 1980s have confirmed the presence of schistosoma eggs and adult worms in upper and lower female genital organs [7-12]. The relation of schistosomiasis to cervical cancer has only rarely received attention [13,14]. Cervical cancer is the most common malignancy among women in African countries [15]. Since diagnosis and treatment are inadequate or non-existent even in tertiary hospitals, women often present at a hospital in an advanced and desolate stage of the disease [16,17].

Tanzania belongs to the poorest countries in the world [18]. Its health care has to struggle with an overwhelming burden of diseases relying on a weak infrastructure. Infectious diseases are prominent, notably so sexually transmitted ones. STIs, including HIV, have already been seen in primary school-children, although at a low level [19]. At least one ongoing treatable reproductive tract infection was present in 64 percent of women attending urban primary healthcare facilities in the Northern Region and in 39 percent of women attending antenatal care in rural health care facilities [20,21]. Professional knowledge and awareness of female genital schistosomiasis can bring a lot to the quality of care in sexual and reproductive health.

In this article we present evidence of female genital schistosomiasis in referred specimens to a pathology department in Northern Tanzania.

## Methods

The Kilimanjaro Christian Medical Centre (KCMC) is the consultant hospital for over 12 to 15 million inhabitants of Northern Tanzania. It is also the Medical College of the Tumaini University. The Department of Pathology receives biopsies and surgical specimens from over 50 hospitals all over the country, which means that they are responsible for about 35 percent of this type of examinations made in Tanzania. Around 5,000 specmines of all kinds are processed yearly.

The specimens are routinely fixed and preserved in 10 percent formaldehyde for transportation. At KCMC the tissues were paraffin-embedded. Five to 7 μm thick sections are cut on a microtome and mounted onto microscope slides. The sections are stained with Hematoxylin and Eosin for the microscopical examination. Two slides are prepared and analysed from each paraffin-embedded block.

All surgical specimens sent to or taken at KCMC between 1994 and 2005 in which the histopathological diagnosis of schistosomiasis was established were reviewed. Age, provisional diagnosis and symptoms, when available, as well as all specific histopathology information were noted for the female patients where the examination had shown the presence of schistosomiasis.

### Ethical considerations

This study is part of a larger project "Sustainable Prevention of Endemic Schistosomiasis" (SPES), a research collaboration between KCMC and the Skaraborg Institute for Research and Development, Skövde, Sweden. Ethical clearance was given by the Research and Ethical Committee of the KCMC, Tumaini University, Tanzania. Research data were processed and stored anonymously.

## Results

During the study period (12 years) 423 organ specimens examined at Department of Pathology (equals 0.7% of the total number of specimens during this time period) had a histopathologically confirmed schistosomiasis diagnosis. In 172 cases (40.7%) schistosomiasis was diagnosed in bladder and/or urethra and/or urether specimens, in 15 cases (3.5%) in liver specimens, in 45 cases (10.6%) in intestine specimens (jejunum, colon, appendix, rectum) and in 8 cases (1.9%) in mesentery/omentum. Genital schistosomiasis was diagnosed in 176 specimens from genital organs (41.6%: 115 organ specimens from female patients). Totally, genital specimens from 111 women were examined. The age of the patients ranged from five to 61 years, with a median age of 34 years. The specimens were sent from 24 hospitals in 13 regions of mainland Tanzania. Organs represented were peritoneum, uterus, cervix, ovary, Fallopian tubes, vagina/vulva, perineum and labia. The specimens consisted of biopsies from in situ and removed organs. The provisional diagnosis on the referral form was mostly a suspicion of malignancy. The main symptoms reported were bleeding disorders (48%), ulcer (17%), tumor (20%), lower abdominal pain (11%), and infertility (7%).

Eleven percent of the specimens came from women less than 20 years old. Table 1 shows the age distribution per organ examined. In 84 patients (75%) no other diagnosis besides schistosomiasis was established.

**Table 1:** Number of examined organs per age group of 105 of the 111 female patients for whom age information was available. Percentages in brackets.

Specimen^a^	Agegroup (years)			
	5 – 19	20 – 29	30 – 39	≥ 40
	
Cervix	2 (20)	15 (47)	18 (50)	28 (74)
Uterus	1 (10)	5 (16)	5 (14)	7 (18)
Ovary		5 (16)	7 (19)	2 (5)
Tubes		2 (6)	2 (6)	1 (3)
Vagina/Vulva		1 (3)		
Labia^b^	5 (50)			
Skin/Perineum	2 (20)			
Peritoneum		4 (13)	4 (11)	
Total	10	32	36	38

^a ^double registration possible.

^b ^age range: 8 – 18 years.

Schistosomiasis was seen in simultaneously in two sites in 13 patients and in three sites in two patients with the following combinations, cervix and uterus; uterus and peritoneum; ovary and Fallopian tube; ovary and peritoneum; uterus, cervix and ovary. In five specimens schistosome eggs as well as adults worms were detected (labia, cervix, and ovary).

### Specimens from the cervix only or in combination with other organs

Of all the 71 cervical specimens 14 (20%) showed signs of malignancy; epithelial dysplasia (4), carcinoma in situ (2), squamous cell carcinoma (6), adenocarcinoma (1) and lymphangiosis carcinoma (1). In the rest schistostosomias associated with chronic inflammation was diagnosed.

For the 62 patients in this group symptoms reported were intermenstrual bleeding, post-coital bleeding, irregular menstruation, contact bleeding, and post-menopausal bleeding, with a median duration of symptoms given as four months (range 2 – 96 months). The clinical findings were described in 31 patients as suspicious appearance of the cervix (61%), ulcer/erosion (35%), growth/polyp (16%), tumor (13%), and friable/hard/nodular cervix (13%).

The median age of the women with or without cervical malignancy was 39 years.

The verification of a provisional carcinoma diagnosis was explicitly requested for 53 patients (Table 2). The carcinoma diagnosis could be confirmed for 13 of them.

**Table 2:** Results of the histopathology examination for patients with the provisional diagnosis of carcinoma on the request form.

Specimen	Provisional diagnosis – carcinoma	Histological diagnosis	
		Carcinoma and schistosomiasis	Schistosomiasis only
Cervix			
Only cervix	47	12	35
Cervix and uterus	4		4
Cervix and endometrium	1		1
Cervix and vagina	1	1	
Ovary			
Only ovary	4	2	2
Ovary and uterus	1		1
Ovary and Fallopian tubes	1		1
Ovary and peritoneum	1		1

### Specimens from uterus/endometrium

Three of the ten uterine specimens showed only severe schistosomiasis. Schistosomiasis was associated with leiomyoma in four specimens, and in one specimen each with adenomyosis, adenomyosis/leiomyoma, and endometritis. Seven patients had undergone total hysterectomia, in one case with right salpingo-oophorectomia. One patient (28 years) had a hysterectomia performed due to a tumor of the cervix, however, the histopathology diagnosis of this case was severe schistosomiasis of the cervix and uterus. In one patient a squamous epithelial carcinoma associated with schistosomias was diagnosed in the bladder, the right and left ureter, and the uterus. In seven specimens of the endometrium, schistosomiasis was diagnosed; in one of these cases concomitant tuberculosis could not be ruled out.

### Fallopian tubes/ovary/peritoneum

This group consisted of 14 samples from ovarian tissue and five from the Fallopian tubes. Severe scarring due to schistosomiasis was seen in one case and in two cases there was a specific inflammatory reaction. In three cases schistosomiasis of the peritoneum, in one schistosomiasis of pelvic tissue, and in one schistosomiasis of a lymphnode was also noted. Infertility was reported in four patients where tubal schistosomiasis was found (21, 25, 31 and 33 years old). A provisional diagnosis of cancer was confirmed in two out of seven women (Table 2).

### Labia/vulva/perineum

Schistosomiasis of the external genital organs was only seen in girls and one young woman: vulva (1 case: 8 years old), perineum (2 cases: 5 and 6 years old) and labia (5 cases: 10, 11, 13, 13 and 18 years old). Labial tumors were reported in the request form in four cases, in two cases associated with ulcera and verrucous skin.

## Discussion

Schistosomiasis is an important and highly prevalent helminthic infection related to water contact and poverty and is affecting approximately 250 million people living in the tropical and sub-tropical parts of Sub-Saharan Africa, Asia and South America [2]. The number of women with schistosomiasis-related signs and symptoms thus ought to be high and could easily concern millions. Re-assessing the world-wide clinical morbidity caused by schistosomes, however, van der Werf et al. [22] have not included the clinical picture of genital schistosomiasis in the analysis because of insufficient data. Since the first description more than 100 years ago, numerous case reports published over decades in journals of different specialities (gynaecology, pathology, urology, histology, tropical medicine) have presented clinical features – often described as unusual – of genital schistosomiasis in female patients. In 1949 Charlewood concluded by examining reports of the South African Institute of Medical Research: "...it is apparent that practically all gynaecologists who have been practising in Johannesburg and Durban for any length of time have encountered gynaecological manifestations of Bilharzia. Yet, it is surprising how meagre and unsatisfactory is the literature on the subject" [23]. The situation has not changed. Systematic research on the morbidity caused by genital involvement during the course of a schistosome infection is so far sketchy and research on the possible impact of genital schistosomiasis on viral co-infections is to our mind virtually non-existent.

Retrospective histopathology studies carried out in Egypt, Malawi, Mozambique, South Africa, Tanzania and Zimbabwe have shown that genital involvement mainly caused by Schistosoma haematobium most often occur in the vagina and cervix, less frequently in the ovary, tube and uterus. Gelfand et al. [24] found in a series of consecutive autopsies, using the sensitive digestion method, schistosome eggs in female genital organs in 58 percent of the cases. Investigations based on specimens from gynaecological surgical interventions (biopsies, curettage, organ exstirpation) can give information about the relative frequency of pathological changes in the different genital organs. In Gabon, Mozambique, Malawi and Madagaskar female genital schistosomiasis has been diagnosed in from 0.7 percent (Madagascar) up to 29 percent (Mozambique) of such genital specimens [11,12,25,26]. In cases of ectopic pregnancy, tubal schistosomiasis was found in Cote d'Ivoire and Gabon in 3.6 and 1.8 percent, respectively [25,27]. On Pemba Island in Tanzania tubal schistosomiasis was diagnosed in 39.8 percent of all cases with primary or secondary infertility [28]. In Cote d'Ivoire schistosomiasis of the placenta was diagnosed in a prospective study in 22.3 percent of 322 cases. When a sub-group of 87 of patients were examined for urinary schistosomiasis, in only 12.6 percent of the women the diagnosis of urinary schistosomiasis could be confirmed [8].

Out of the 423 cases with a histopathological confirmed diagnosis of schistosomiasis the genital and urinary involvement occurred to the same degree (41% and 42%). In no case a provisional diagnosis of female genital schistosomiasis was mentioned on the request forms. Whereas six percent of the cases were due to multi-focal infections in Malawi [11], we found involvement in more than one organ in 12 percent.

Genital schistosomiasis is associated with bleeding disorders, lower abdominal pain but also with infertility. A simultaneous affection of the ovaries and Fallopian tubes and/or peritoneum, as was found in our material, has been reported previously [29-31]. A high tissue egg burden is associated with generalized inflammation and fibrosis and thus mechanically impairs the tubal motility or the tubal patency [32-34]. Ectopic pregancies or infertility are the highly possible outcomes of this [28,35-38].

**Figure 1:**
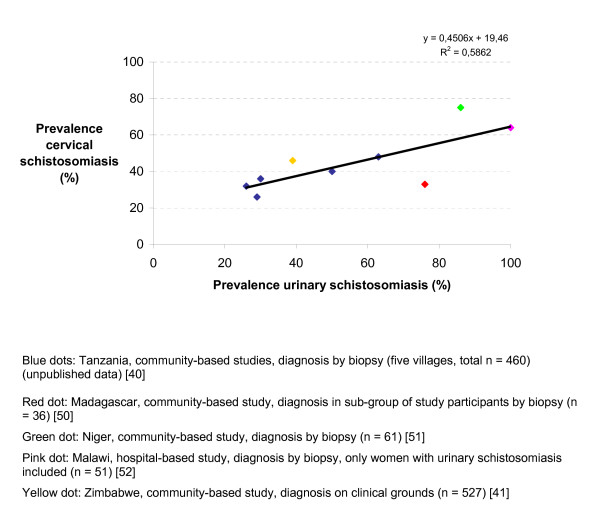
Combined analysis of data from five schistosomiasis-endemic countries. Relation between prevalence of urinary schistosomiasis and the occurrence of genital schistosomiasis of the cervix uteri in women with urinary schistosomiasis.

Cervical schistosomiasis as a chronic disease persisting for years causes damages of the cervical epithelium and can theoretically render the local immune environment favourable for viral infections such as caused by HIV and human papilloma virus (HPV) [5,6]. Impaired cell-mediated immunity has been shown in patients with schistosomiasis of the urinary bladder associated with carcinoma of the bladder or of the prostate [39]. Clinical evidence corroborates this hypothesis. Firstly, according to cross-sectional studies carried out in schistosomiasis-endemic areas cervical involvement is common. Secondly, analysing prevalence data so far available, one can see a correlation between prevalence of urinary schistosomiasis and cervical schistosomiasis; the higher the prevalence of urinary schistosomiasis the higher the prevalence of cervical schistosomiasis (Figure 1). Thirdly, cervical schistosomiasis is associated with lesions such as sandy patches, erosion, mucosal bleeding, and abnormal blood vessels [40,41]. The clinical features seen in the patients of our study were cervical tumors, friable, easily bleeding cervix, and suspect appearance of the cervix. Berry [42] in an extensive series of histopathological investigations of cervical specimens, noted presence of eggs without inflammation; mild cervicitis with scattered lymphocytes, histiocytes and plasma cells, sometimes associated with atrophic epithelium; scattered eosinophils throughout the connective tissue; interstitial hemorrhage, chronic cervicitis often with ulceration. Lesions in the cervix impair the epithelial integrity and thus make it biologically plausible that schistosomiasis can increase the risk for transmission of HIV-infection in the same way as is seen in other genital ulcer disease [43]. This could of course be valid for other infectious agents with the same transmission pattern as HIV. Another memento is that the mucosal changes by schistosomiasis could already be there before the young women starts their sexual-reproductive life.

The possible association between cervical cancer and cervical schistosomiasis have been studied retrospectively by histopathology analysis. Genital schistosomiasis was diagnosed in 1.7, 1.9 and 3.0 percent of cases with cervical cancer in three studies from Malawi and Tanzania [11,13,14]. In all the studies the percentage of cervical schistosomiasis detected in cervical tissue without cervical cancer was higher (4.3%, 6.0%, 9.8%). A possible explanation for these findings is the fact that the clinical features of cervical schistosomiasis simulate those produced by malignancy. The results of our study point in this direction: the provisional diagnosis of cervical cancer in patients with genital schistosomiasis could be confirmed in only 20 percent of the patients. As a consequence women might undergo surgery such as hysterectomy on false clinical premises, which we saw in our study. The studies carried out so far cannot support the hypothesis of cervical schistosomiasis being a co-factor to HPV infection for the development of cancer. It has, however, been brought forward that chronic cervical inflammation and parasitic infection impairing the cellular immunity might contribute to the high prevalence of HPV types other than HPV16 seen in sub-Saharan Africa [44]. Petry et al. [45] showed that the HPV prevalence of patients orignating from endemic areas in Tanzania and from German controls were similar (34.5% versus 26.9%), however, women with a history suggestive for schistosomiasis and/or active schistosomiasis had a higher risk for habouring high risk HPV types. The authors concluded that the infection with S. haematobium sems to favor persistent genital HPV infection either by traumatizing the general epithelium and/or by local immunosuppression. Additionally, the interplay of HIV, schistosomiasis and HPV might very well have an impact on HPV infection. Reporting two cases of HIV-positive women with schistosomiasis of the cervix Prabhakaran and Brown [46] proposed timely treatment against schistosomiasis to prevent aggressive HPV infection and its consequences in HIV-infected patients. The authors argue that persistent schistosomiasis may result in earlier HPV relapses because schistosomiasis increases the likelikhood of STDs. Recently, two cases of cervical schistosomiasis and squamous cell carcinoma have been reported in which high-risk HPV could not be detected [47], however, with the low number of specimens the possibilty of false negative test results and/or insuffienct conservation have to be considered as alternative explanation [48]. Another possible patho-mechanism might be the inactivation of tumor suppressor genes. Mutations of the p53 tumor suppressor gene which encodes a protein involved in the growth and regulation of cells and of components of the DNA damage control response is one of the most frequent genetic alteration in a variety of malignancies. Mutations in the tumor suppressor gene p53 have been observed more frequently in patients with schistosomiasis-associated bladder cancer compared to patients with non-schistosomiasis associated bladder cancer [49].

## Conclusion

The retrospective histopathological data presented give an overview of the occurrence of schistosomiasis in genital organs in women living in a schistosomiasis endemic area. Conclusions on the prevalence of female genital schistosomiasis in the different genital organs can not be drawn from this type of study. However, the results indicate that genital schistosomiasis has to be considered as differential diagnosis in a schistosomiasis endemic areas. Genital schistosomiasis misdiagnosed as cancer can be detrimental and devastating, if surgery is done. Furthermore, cervical genital schistosomiasis misdiagnosed as an STI can lead to ineffective treatment. Schistosome-, HIV- and HPV-infections are highly prevalent in sub-Saharan Africa, indications that cervical schistosomiasis can influence the course of STIs are at hand.

## Competing interests

The author(s) declare that they have no competing interests.

## Authors' contributions

BS participated in the collection of data, interpretation fo findings and the drafting of the manuscript. GP participated in the analysis, the interpretation, the presentation of findings and the preparation of the manuscript. SM participated in the interpretation of the results. IK participated in the interpretation of the findings and the preparation of the manuscript. All authors read and approved the final manuscript.

## Pre-publication history

The pre-publication history for this paper can be accessed here:


